# On the effect of protein conformation diversity in discriminating among neutral and disease related single amino acid substitutions

**DOI:** 10.1186/1471-2164-13-S4-S5

**Published:** 2012-06-18

**Authors:** Ezequiel Juritz, Maria Silvina Fornasari, Pier Luigi Martelli, Piero Fariselli, Rita Casadio, Gustavo Parisi

**Affiliations:** 1 Departamento de Ciencia y Tecnología, Universidad Nacional de Quilmes, Buenos Aires, Argentina; 2 Biocomputing Group, Department of Biology, University of Bologna, Italy; 3 Biocomputing Group, Department of Computer Science, University of Bologna, Italy

## Abstract

**Background:**

Non-synonymous coding SNPs (nsSNPs) that are associated to disease can also be related with alterations in protein stability. Computational methods are available to predict the effect of single amino acid substitutions (SASs) on protein stability based on a single folded structure. However, the native state of a protein is not unique and it is better represented by the ensemble of its conformers in dynamic equilibrium. The maintenance of the ensemble is essential for protein function. In this work we investigated how protein conformational diversity can affect the discrimination of neutral and disease related SASs based on protein stability estimations. For this purpose, we used 119 proteins with 803 associated SASs, 60% of which are disease related. Each protein was associated with its corresponding set of available conformers as found in the Protein Conformational Database (PCDB). Our dataset contains proteins with different extensions of conformational diversity summing up a total number of 1023 conformers.

**Results:**

The existence of different conformers for a given protein introduces great variability in the estimation of the protein stability (ΔΔG) after a single amino acid substitution (SAS) as computed with FoldX. Indeed, in 35% of our protein set at least one SAS can be described as stabilizing, destabilizing or neutral when a cutoff value of ±2 kcal/mol is adopted for discriminating neutral from perturbing SASs. However, when the ΔΔG variability among conformers is taken into account, the correlation among the perturbation of protein stability and the corresponding disease or neutral phenotype increases as compared with the same analysis on single protein structures. At the conformer level, we also found that the different conformers correlate in a different way to the corresponding phenotype.

**Conclusions:**

Our results suggest that the consideration of conformational diversity can improve the discrimination of neutral and disease related protein SASs based on the evaluation of the corresponding Gibbs free energy change.

## Background

Human single nucleotide polymorphisms (SNPs) are the most frequent type of genetic variation in humans. Less than 1% variations are associated with non-synonymous coding SNPs (nsSNPs). About 64,971 nsSNPs are presently listed as human polymorphisms and disease single amino acid substitutions, SASs, (http://www.uniprot.org/docs/humsavar) and approximately 40% of these SASs are disease related.

It has been documented that in proteins a single amino acid substitution (SAS), can produce the loss of function in different ways. Although the less frequently found [1], the most obvious mechanism at the disease origin is due to change of key residues participating directly in protein function. This is the case when residue substitution occurs at the active site or in binding-sites for substrate and/or allosteric regulators [2-4]. When the biological functional unit is a complex, SASs at the subunit interface may also hamper the activity [4,5]. A second possible mechanism is related with the perturbation of protein stability. Residue substitution can indeed destabilize the native protein fold [1,6]. Also stabilizing residue changes have been reported to be associated with diseases [7,8]. Furthermore, related with protein stability alteration, the origin of pathogenesis was also related with anomalous post-translational modifications [9] and aggregation [10]. The correlation among protein SASs and their involvement in human diseases has been proven to be moderate [11], suggesting that change in protein stability is not the only source of diseases.

Protein stability can be estimated measuring the variation of Gibbs free energy (ΔΔG) between the folded and unfolded state of the protein. Most of the experimental data reported in literature are contained in ProTherm [12], a thermodynamic database of proteins and their variation in different organisms. Alternatively, several computational methods have been developed to estimate stability changes caused by substitution of lateral side chains in proteins (ΔΔG=ΔG_wild_-ΔG_mutated_). Most of them rely on the analysis of the energetic and/or structural perturbation introduced by the variations in the protein native structure. Although computationally intensive, early methods used all atom models to estimate ΔΔG [13]. Soon later, simplified potentials coupled with limited conformational searches [14,15] and the use of different types of potentials, like those based on hydrophobic interactions [16], secondary structure [17], inter-residue contacts [18] and knowledge-based [19], allowed to study the effect of different mutations in proteins in a reasonable computational time. Recently machine learning based approaches have been implemented for the prediction of ΔΔG in proteins upon residue substitution taking as input either the protein structure or sequence (for a recent review see [20]). The discrimination among disease related and neutral SASs can be investigated by determining ΔΔG upon residue change in the protein. This analysis is based on the notion that most harmful SASs are related to protein stability perturbation above a certain threshold ΔΔG (±1 kcal/mol). Most methods suited to predict free energy changes have been recently benchmarked in relation to their ability to discriminate disease from neutral SASs based on the corresponding ΔΔG value and their performance has been proven to be rather poor [20].

In most methods, predictions of SASs effects are commonly estimated using a single structure of the corresponding protein [21-24]. This approach apparently underestimates the well established concept that the native state of a protein is better represented by an ensemble of conformers [25-27]. The conformational ensemble is a key concept to explain essential properties of proteins like function [28-30], enzyme and antibody promiscuity [31,32], enzyme catalytic power [33], signal transduction [34], protein-protein recognition [35] and the origin of new functions [36]. Conformers describing the native state of a protein exist in a dynamic equilibrium which changes in response to the presence of ligands such as substrate or allosteric modulators that shift the relative conformational population [37,38]. From a practical perspective, conformational diversity could be described using experimentally available structures of the same protein obtained in alternative conditions. As these different structures for the same protein have been obtained under different conditions (for example presence of substrate, inhibitors or allosteric activators) they can be taken as snapshots of protein dynamics and then characterize putative conformers belonging to the native ensemble [39,40]. In this way, the description of the native ensemble of the protein will be more or less complete depending on available experimental data. A way to describe the extension of conformational diversity could be the estimation of the maximum RMSD measured between the available conformers. The distribution of the structural diversity extension measured in this way in the protein space was recently studied [41]. The analysis involves an all vs. all comparisons between structures of the same sequence deposited in PDB database [42]. Conformer distribution exhibits a peak at 0.3 Å RMSD with a large skew that ends at about 24 Å RMSD.

In the present work we investigated how the presence and extension of conformational diversity affect the estimation of ΔΔG in proteins with neutral and disease related SASs. Like protein function relies on the existence and preservation of the dynamic ensemble of conformers, the study of the effect of a SAS in each conformer of a given protein could help to understand the loss of function. We used a set of 803 SASs (482 disease related and 323 neutral) in 119 proteins showing different extension of conformational diversity. These proteins were taken from the Protein Conformational Database (PCDB) [43] a redundant collection of protein structures linked with biological information. ΔΔG for each SAS in each conformer for a given protein was estimated using FoldX [44]. We found that the ΔΔG estimated value for a SAS highly depends on the conformer used in the estimation. In 35% and 58% of the studied proteins we found that at least one SAS could be classified as neutral, stabilizing or destabilizing depending on a ΔΔG threshold value of ±2Kcal/mol and ±1 Kcal/mol respectively. We also found that the consideration of conformational diversity increases the performance of the prediction of disease related SASs based on ΔΔG analysis. Our results show that the different conformers correlate in different ways with the phenotype (disease or neutral) and that, in most cases, one conformer per protein correlates perfectly with the corresponding phenotype. Our results indicate that the use of conformational diversity may be important to understand the effects of neutral and disease related SASs on protein stability.

## Results

### Extension of protein conformational diversity

**Figure 1:**
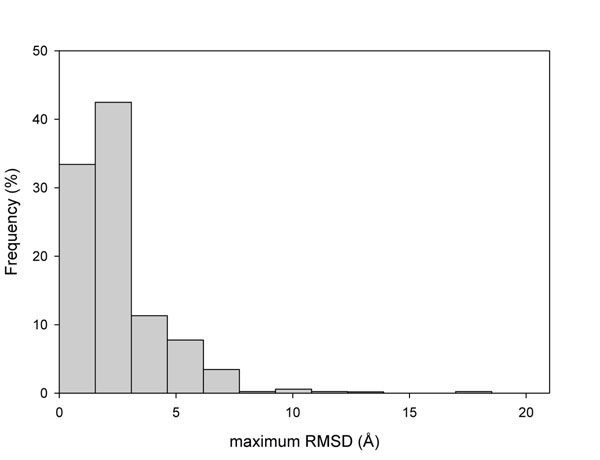
Distribution of maximum RMSD between conformers corresponding to the 119 proteins in the dataset used in this study.

**Figure 2:**
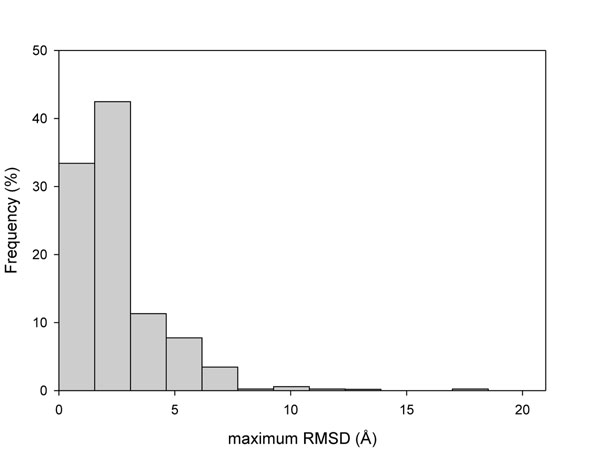
Distribution of ΔASA (Å^2^) for substituted positions derived from the analysis of the conformational ensemble for each of the 119 proteins in the dataset.

The 119 proteins studied in this work were linked to the PCDB database [43]. All conformer coordinates contained in PCDB for each protein were derived from the PDB database (http://www.rcsb.org). The structures were obtained under different conditions, mainly in the presence of different ligands that shift the population equilibrium of the different conformers in the ensemble [37,45]. Our dataset has an average maximum root mean squared deviation (RMSD) between conformers of 2.51 Å and an average number of conformers per protein of 8.6. The distribution of the maximum RMSD (the maximum RMSD displayed between all conformers of a given protein) is shown in Figure 1. Considering that the average RMSD for a protein crystallized under the same condition ranges from 0.1 and 0.4 Å [42] and from the distribution shown in Figure 1, we concluded that our dataset contains proteins with moderated to extreme conformational diversity (for details on conformational diversity per protein see additional file 1). We also computed the relative accessible surface area (ASA) of the positions involved in SASs (neutral and disease related) as described in Methods. We found changes in the maximum Δ(ASA) between conformers, with a maximum value of 98.6 Å^2^ and an average value of 12.0 Å^2^ (Figure 2). This distribution reflects the structural changes at the SAS positions between conformers. In fact, using Δ(ASA) values, 33% of the proteins have at least one position that, depending on the chosen conformer, can be classified as buried (ASA<20Å^2^) or solvent exposed (ASA>20Å^2^). Previous observations suggested that ΔΔG values upon residue substitution inversely correlate with the corresponding ASA values [46]. We therefore can expect large variations in the ΔΔG estimation upon changes on the different conformers considering the large ASA variation.

### Variation of ΔΔG estimation using conformational diversity

**Figure 3:**
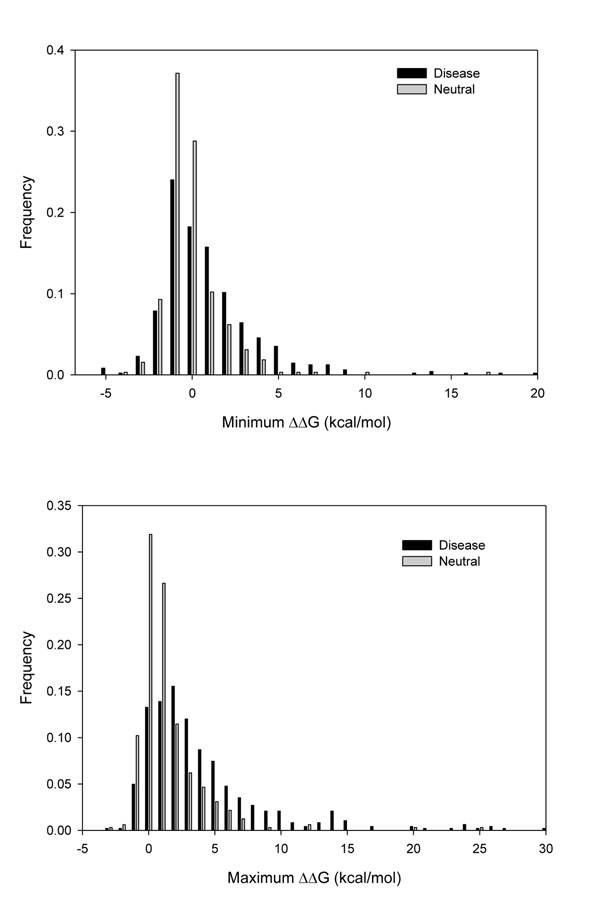
Distributions of maximum and minimum values of ΔΔG obtained for the different conformers for each protein in the dataset. Disease and neutral SASs are shown as separate distributions.

**Figure 4:**
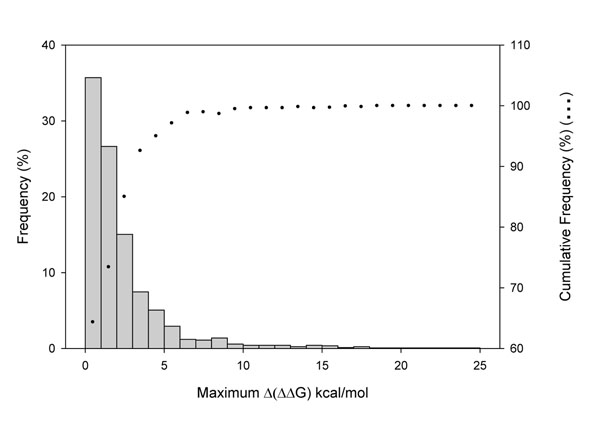
Distribution of maximum Δ(ΔΔG)(kcal/mol) for the 803 SASs studied. Bars represent the frequency and dots the cumulative frequency.

For each of the 803 SASs a ΔΔG estimation was performed for all the conformers of each protein using FoldX [44]. The accuracy of FoldX to predict stability changes has been discussed before [20,46]. For each mutation we registered the maximum and minimum ΔΔG values and the maximum difference of the ΔΔG values among different conformers of the same protein (maximum Δ(ΔΔG)). The distributions of maximum and minimum ΔΔG values and the distribution of the maximum difference of ΔΔG values (max. Δ(ΔΔG)) are shown in Figures 3 and 4, respectively, for both disease related and neutral SASs. We found that maximum and minimum values of ΔΔG of disease related SASs have higher (destabilizing) values compared with those of neutral SASs. The distributions of minimum ΔΔG values have average values of 1.47kcal/mol and of 0.38 kcal/mol for disease related and neutral SASs, respectively. This distribution difference is significant (Kolmogorov-Smirnov test with P-value < 1 10^-5^). In turn, average maximum ΔΔG values for disease and neutral SASs are 4.63 and 1.86 kcal/mol respectively (Kolmogorov-Smirnov test with P-value < 1 10^-5^). Considering the distributions of maximum variation of the Δ(ΔΔG), most of the values (69%) are below 1kcal/mol. This value can be regarded as a typical standard error in the estimation of ΔΔG [44] (Figure 4). However, 31% of the SASs have maximum Δ(ΔΔG) above the standard error and a significant difference between the ΔΔG estimations of the different conformers.

When each SAS is mapped into its corresponding protein, we found that in 35% of the cases there is at least one SAS that can be classified as neutral and stabilizing or neutral and destabilizing depending on the considered conformer. It is interesting to note that for this 35%, the average of the maximum RMSD between conformers is 3.78 Å compared to 2.38 Å for proteins without ambiguous predicted stability changes. The discriminative threshold of ΔΔG was set at ± 2 kcal/mol, as it was previously discussed by Worth and coworkers (Worth, Preissner, and Blundell 2011). However, other works have defined different thresholds to classify the stability changes of a SAS [47-49]. When we analyzed the data distribution for ΔΔG values using a threshold of ±1 kcal/mol, the number of proteins with at least one ambiguous prediction was even higher (58%) (these calculations can be done using the data and information included as additional file 1). Thus, the extension of conformational diversity measured by the maximum RMSD between conformers increases the variability of the stability prediction of SASs generating certain ambiguity in the prediction. It is noteworthy that this ambiguity is manifested in the same proportion respect to the total population of SASs in disease-associated SASs as in polymorphic SASs. That is, the proportion of neutral SASs with respect to the total number of SASs is similar to the proportion of neutral SASs with ambiguous prediction with respect to the total number of SASs with ambiguous predictions (almost 29%). From this we can conclude that the uncertainty in the evaluation of the thermodynamic effect of a SAS equally affects neutral and disease related SASs.

**Figure 5:**
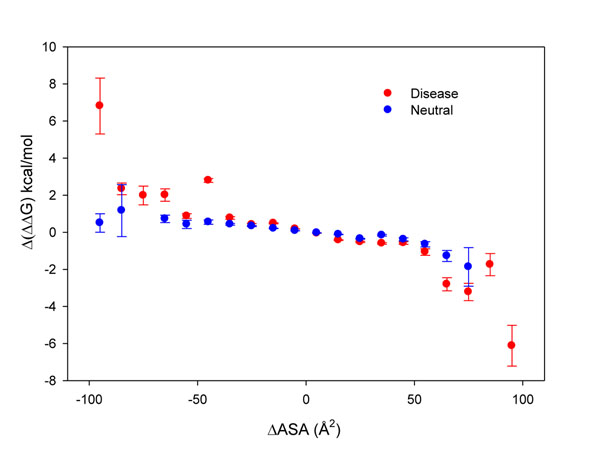
Scatter plot of average differences of Δ(ΔΔG) (kcal/mol) among conformers as a function of averaged ΔASA (Å^2^) and their respective error bars. Dots represent the average derived from ranges of 10 units of ΔASA and are represented in the centre of each interval.

In order to explore the effect of changes in ASA among conformers, we compared Δ(ΔΔG) and ΔASA values for disease and neutral SASs (Figure 5). It is interesting to note that at decreasing ΔASA (<0), Δ(ΔΔG) values indicate protein destabilization for disease related SASs (Δ(ΔΔG)>2kcal/mol) and remain unaffected in the case of neutral SASs (Δ(ΔΔG)<2kcal/mol). On the contrary, when positions become more exposed to solvent (ΔASA >0) the ΔΔG turns to stabilizing values. However, in this last condition, the neutral and disease related SASs present almost the same behavior. Most of the values between ±50 Å^2^ of ΔASA (64%) involve differences between exposed positions which explains the low variation in Δ(ΔΔG) (approximately ±2kcal/mol) as it is derived from the analysis of the distributions shown in Figure 5.

Summing up, all the observations indicate that there is a large spread of estimated ΔΔG values for the different conformers due to their structural differences. The spread and, eventually, the corresponding ambiguity in the prediction of ΔΔG, can blur the correlation among ΔΔG values and the SAS type (disease related or neutral) when only one protein structure is used, as it is routinely the case.

### Prediction of disease related SASs using ΔΔG

In order to explore how well the explicit consideration of conformational diversity could improve the estimation of disease related variants using stability measurements, we calculated the Mathews Correlation Coefficient (MCC) among ΔΔG computed values and the classification of the SASs as disease related or neutral. When conformational diversity is taken into account for each protein, a given SAS will result in a different number of ΔΔG estimated values (the number of values is equal to the number of conformers belonging to this protein). We first calculated MCC for all the data (14297 ΔΔG values for 119 proteins with an average number of conformers per protein of 8.6). Secondly, we characterized the change in the stability after a SAS using the minimum, maximum and average of the ΔΔGs obtained from the set of corresponding conformers for a given protein and a given SAS. Finally, to contrast our hypothesis (that could be convenient to consider the conformational diversity) we also estimated MCC using a random selection of ΔΔG values derived from those obtained for each SAS in the set of corresponding conformers. The results, including the corresponding sensitivity, specificity and accuracy values are reported in Table 1 and indicate a significant difference between MCC corresponding to maximum values per conformer compared to random (P-value= 0.02). Random gives an estimation of the performance of the use of the stability changes to predict disease related mutations using only one structure per protein in our dataset. Our results for random selection of single ΔΔG values agree with the performance reported in recent works [47]. The higher values of MCC using the maximum ΔΔG agree with the averaged maximum values for disease related (4.62 kcal/mol) and neutral SASs (1.86kcal/mol) shown in Figure 3. All the results are in agreement with previous work highlighting difficulties to discriminate disease related SASs on the basis of protein stability criteria [24].

**Table 1:** Scoring the capability of discriminating among disease related and neutral SASs on different set of conformers.

	MCC	Accuracy	Specificity	Sensitivity
Global*	0.19	0.54	0.76	0.44
**Minimum°**	0.23	0.54	0.85	0.34
**Maximum°**	0.36	0.68	0.69	0.68
**Average°**	0.25	0.62	0.80	0.5
**Random^**	0.25	0.60	0.68	0.55

*Global uses all data neglecting the correspondence between proteins and their conformers. °Minimum, Maximum and Average characterize the values of ΔΔG per SAS taking into account the conformers for each protein. ^Random MCC was calculated as an average over 50 independent selections of a randomly taken one ΔΔG value per SAS and per protein. In all the cases the threshold is ± 2kcal/mol. The difference between MCC values for Maximum and Random is significant at P-value=0.02. For definition of the different scoring indexes see text.

**Figure 6:**
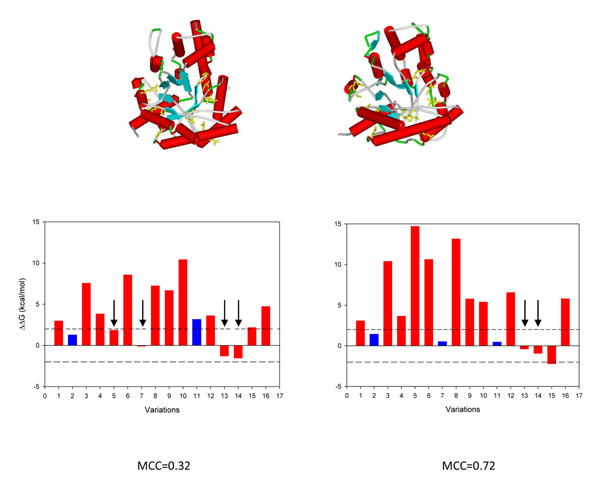
Example showing how conformational diversity affects ΔΔG estimation and MCC values. The protein fructose-biphosphate aldolase B (P05062) is shown in the example. Two conformers were found for this protein with a RMSD = 1.3 A. At the top of the figure there are cartoon representations of the two conformers with the mutated amino acids represented in stick (in yellow). Bars graphics represent ΔΔG values (red for disease associated mutations and blue for neutral mutations) and finally the corresponding MCC for each conformer. Black arrows indicate wrong predictions based in the reference interval of ±2kcal/mol. As structural changes produce variations in ΔΔG the different conformers have different MCC values.

As a final consideration, we observed that for each SAS in a given protein, and considering all conformer derived ΔΔG values, in 763 out of 803 SASs (94.8%) there is at least one conformer whose ΔΔG value correlates perfectly with the disease or neutral phenotype (at a given/fixed ΔΔG discriminative threshold). For some proteins in our dataset, well populated with disease as well as with neutral SASs (25 proteins), we computed the conformer specific MCC to find the best conformer associated to disease or neutral phenotype. Interestingly most of these proteins show a different extent of variability of MCC values for the corresponding conformers. MCC values range from 0.67 to 1 for adenine phosphoribosyltransferase (P07741, with 4 conformers and 5 disease related and 1 neutral SASs), from 0.37 to 0.72 for fructose-biphosphate aldolase B2 (P05062, 2 conformers with 13 disease related and 3 neutral SASs) from 0.068 to 0.33 for the uroporphyrinogen decarboxylase (P06132, 7 conformers, 34 disease related and 6 neutral SASs) and from -0.04 to 0.27 for the transthyretin (P02766, 71 conformers, 74 disease related and 9 neutral SASs). In Figure 6 we show an example of how conformational diversity affects the estimation of ΔΔG and their correlation with disease. Cartoon representations for the two conformers considered for fructose-biphosphate aldolase B (P05062) are shown at the top; the central panel includes the values of ΔΔG and at the bottom the corresponding MCC. Structural changes among conformers promote different local arrangements of variants resulting into different ΔΔG values. It is then expected that different conformers correlates in a different way with the occurrence of disease or neutral SASs. In order to obtain a further understanding on the role of the different conformers and the occurrence of disease or neutral SASs, we have mapped the occurrence of ligands in each conformer to detect bound and unbound states for each protein. We found that in 69% of the proteins the conformer with the maximum ΔΔG corresponds to the bound state of the protein. However this result should be taken with care. As explained in the Methods section we have detected the presence of ligands using Procognate database. In this procedure we just detected the presence of ligands that can be substrates, inhibitors, cofactors or allosteric effectors. Since different ligands change the population of conformers in different ways, the “bound” state could contain different conformers per protein. Considering the paucity of conformers available as compared to the protein universe, further work is necessary to completely elucidate their relevance to the disease related phenotype.

## Conclusions

The elucidation of the effect that a single amino acid substitution (SAS) has in a specific phenotype is a central problem in different areas of research. Particularly, in protein computational biology it is very challenging to understand the mechanisms that lead to disease [50]. Estimation of protein stability and its perturbation after single SAS have played a key role to predict the effects of SASs [1,6,11,51]. Notwithstanding important advances in the area, the estimation of stability in proteins is still problematic and its correlation with disease or neutral variants highly depends on the dataset considered [24]. Here, we improved the correlation between the perturbation of protein stability and the classification of SASs as disease related or neutral. This improvement was obtained by considering maximum values of ΔΔG computed taking into account conformational diversity. As the native state is not unique, the conservation of the biological activity of a protein relies on the different conformers of the ensemble. It has been found that the different conformers for a given protein constraints in different way the substitution pattern of proteins [52]. Then it is expected that the different conformers should have different robustness to SASs. Although the correlation is still far from being perfect, our results suggest that the effect of each SAS should be studied in all the protein conformers in order to obtain a better understanding of protein function perturbation and the disease origin. Our results also suggest that conformational diversity could add value to new computational tools for predicting SAS effects and play a key role to obtain a deeper understanding of the relationship among protein structure and function.

## Methods

### Data set collection

A list of proteins with disease and polymorphic SASs was extracted from http://www.uniprot.org/docs/humsavar. This list was linked with PCDB (http://pcdb.unq.edu.ar) database [43]. PCDB contains a collection of redundant protein structures obtained in different conditions (for example presence of ligands, change in oligomerization state, etc.). These structures can be considered as snapshots of protein dynamism, assumption validated by previous works that have proved the correspondence between structural deformations detected under different crystallographic conditions and conformational changes related to the flexibility of the native state [39,40]. The description of the conformational ensemble of the protein native state is limited to the information deposited in PCDB and PDB databases. The maximum RMSD (RMSDmax) between alpha carbon coordinates of the different conformers, calculated with MAMMOTH [53], is taken as a measure of the conformational diversity of the protein. From this cross linking, a dataset containing 119 proteins with different number of conformers was defined (8.6 conformers in average per protein, with a minimum of 2 and a maximum of 73). The length of all the structures in this dataset covers more than 70% of the length of the corresponding protein sequence. The dataset contains 803 SASs with 482 disease related and 323 neutral.

The presence of cognate ligands in each structure was determined using the Procognate database [54]. The cognate ligands deposited in this database are those involved in the biological function of the proteins. After this filtering we found that 35 proteins have bound/unbound states in our dataset.

### Stability and structural measurements

For each SAS and for each protein and conformation, we estimated ΔΔG values using the program FoldX [44]. FoldX uses an empirical potential calibrated to fit in vitro ΔΔG values. Area exposed to solvent was calculated with Naccess program (http://www.bioinf.manchester.ac.uk/naccess/).

### Statistical analysis

To study the performance to predict disease or neutral variants using estimated ΔΔG values, we calculated the accuracy, specificity, sensitivity and also the Matthew’s correlation coefficient (MCC). The equations used were:

A value of MCC=1 defines the best possible prediction, while a value of MCC=-1 indicates the worst possible prediction. A value of MCC=0 corresponds to predictions made by chance.

## Competing interests

The authors declare that they have no competing interests.

## Supplementary Material

Additional file 1 List of SASs mapped on the corresponding structures. List of proteins, structures and corresponding conformers. Click here for file
